# Prediction of intensive care units length of stay: a concise review

**DOI:** 10.5935/0103-507X.20210025

**Published:** 2021

**Authors:** Igor Tona Peres, Silvio Hamacher, Fernando Luiz Cyrino Oliveira, Fernando Augusto Bozza, Jorge Ibrain Figueira Salluh

**Affiliations:** 1 Department of Industrial Engineering, Pontifícia Universidade Católica do Rio de Janeiro - Rio de Janeiro (RJ), Brazil.; 2 Instituto Nacional de Infectologia Evandro Chagas, Fundação Oswaldo Cruz - Rio de Janeiro (RJ), Brazil.; 3 Instituto D’Or de Pesquisa e Ensino - Rio de Janeiro (RJ), Brazil.

## INTRODUCTION

**Figure 1:**
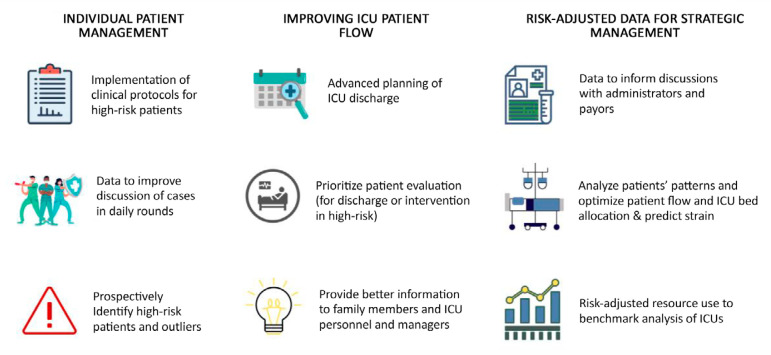
Main benefits of intensive care unit length of stay prediction. ICU - intensive care unit.

The length of stay (LOS) in the intensive care unit (ICU) is one of the most commonly used metrics for quality of care. Despite its potential limitations, ICU LOS is easy to measure, reproducible and can be used as a proxy for resource use, costs, and efficiency.^(1)^ Moreover, it is a patient-centered outcome; therefore, it is of interest to multiple stakeholders, such as patients and families, managers, payors, and ICU personnel (Figure 1). However, in most circumstances, the ICU LOS is used retrospectively to assess ICU efficiency or to understand patients with a long LOS and, thus, elevated costs of care. Therefore, patient LOS prediction at ICU admission could help coordinate care, implement preventive measures, and better communicate with managers, payors, and families, setting realistic expectations.

The ability to predict the LOS for an individual patient could also lead to improved benchmarking, comparing a unique patient LOS with the one for those with similar diagnoses (and, therefore, understanding outliers and identifying targets for quality improvement). It would also allow analyzing the overall results of predicted LOS compared to the observed (real) LOS for all patients (or a subgroup such as sepsis, *acute respiratory distress syndrome*, etc.) in the ICU. This type of analysis (in a similar way as done with mortality through the standardized mortality rates) could be a robust measure of ICU efficiency.^(2)^ The massive amount of data generated in ICUs daily, coupled with recent advances in technology and statistical methods, makes it feasible to develop predictive models that may help clinicians with daily management in the ICU and improve quality of care and efficiency.^(3)^

### What do we know about methods for predicting intensive care unit length of stay?

Most studies that evaluated models for ICU LOS prediction^(4-8)^ used multivariate linear regression and did not test other approaches to compare their accuracy. Linear regression has the advantage of clearer interpretation; however, the assumption of a linear relationship with the covariates is its major limitation. Verburg et al.^(9)^ performed a systematic review in 2017 and concluded that the models developed by these studies did not satisfy general requirements for the prediction of ICU LOS, either to plan resource allocation or to identify individual patient LOS.

We searched the literature to find studies that compared different prediction models for LOS prediction. From inception to October 6, 2020, we searched the MEDLINE, Embase and Scopus databases. The search was limited to the English language and the publication types “article”, “article in press”, and “review”. The search comprised the “title” and “keywords” fields, and no restriction was made for the publication period. We used the following queries: (“ICU” or “Intensive Care”) and (“length of stay”) and (“predict*”). The study selection was fourfold: (i) formulating eligibility criteria; (ii) abstract reading and selection for full-text reading; (iii) full-text reading and selection; and (iv) including new studies by backward and forward search. We considered the following eligibility criteria for study inclusion: studies that included and compared models for ICU LOS prediction, reporting statistics in terms of root mean square error (RMSE), mean absolute error (MAE) or R^2^. Inference studies were not included. We found five prediction studies, and the characteristics of each one are summarized in table 1.

**Table 1:** Characteristics of each prediction study

Author	Cohort	ICU or hospital LOS	Design	Prediction	RMSE	MAE	R^2^ (%)	Log-scaled ICU LOS	Truncation at 30 days	Excluded	Quadratic/cubic terms	Interaction terms	Normalization	Partition	Cross-validation
Moran et al.^(4)^	111.663	ICU LOS	131 mixed-type ICUs	Admission	4.50	2.30	22.0	Yes	No	ICU LOS > 60 days	Yes	Yes	No	N/A	N/A
Verburg et al.^(10)^	32.667	ICU LOS	83 mixed-type ICUs	Admission	7.28	3.43	15.4	Yes	Yes	Hospital LOS > 365 days	No	No	No	Bootstrap	N/A
Houthooft et al.^(11)^	14.480	ICU LOS	14 medical ICUs	Day 5	N/A	1.79	21.9	Yes	No	ICU LOS > 40 days	No	No	Yes (method not informed)	60/40	N/A
Li et al.^(12)^	1214	ICU LOS	One ICU	Admission	0.88	0.87	35.0	Yes	No	No	No	No	Z-score	70/30	10-fold
Muhlestein et al.^(13)^	41.222	Hospital LOS	1,000 hospitals	Admission	0.56*	N/A	N/A	No	No	No	No	No	Z-score	80/20	5-fold
Caetano et al.^(14)^	26.431	Hospital LOS	One hospital	Admission	0.47*	0.22*	81.3	Yes	No	No	No	No	Z-score	N/A	5-fold

ICU - intensive care unit; LOS - length of stay; RMSE - root mean square error; MAE - mean absolute error; N/A - not available;

* Root mean square error/mean absolute error calculated using log transformation.

Verburg et al.^(10)^ compared six regression models to predict the ICU LOS for a dataset of 32,667 ICU admissions. The best models were the generalized linear model (GLM) with a Gaussian distribution and the GLM with a Poisson distribution, and the worst model was the Cox regression. The study tested the ICU LOS log transformation to reduce the skewness and improve the underlying variable distribution symmetry, which presented better results. The authors also tested the truncation at 30 days, which improved the model performance.

Moran et al.^(4)^ compared seven regression models to predict the ICU LOS for a dataset of 111,663 ICU admissions. The best was the linear mixed model (LMM). The authors also tested the log transformation in the ICU LOS, which presented better outputs.

Houthooft et al.^(11)^ compared different data-driven models to predict the ICU LOS for patients remaining in the ICU on day 5. The best performing model was support vector regression (SVR), and the worst was artificial neural network (ANN). The authors included the log transformation of ICU LOS, feature normalization, and feature selection (using the random forest importance list and a backward elimination procedure with SVR) in the preprocessing methodology.

Li et al.^(12)^ created a predictive model using preprocessing techniques, exploratory data analysis, and the least absolute shrinkage and selection operator (LASSO) algorithm. In the preprocessing methodology, the authors included the treatment for missing data, the Box-Cox transformation (for ICU LOS and variables with skewness coefficients greater than 0.5), and Z-score normalization. In the exploratory data analysis step, the study explored new features and the collinearity between existing features.

We also found two prediction articles that compared data-driven models for hospital LOS and presented significant results. Muhlestein et al.^(13)^ developed a novel method to systematically rank, select, and combine different data-driven algorithms, building a model that predicts LOS following craniotomy for brain tumors. The top-performing algorithms were the gradient boosted tree (GBT) and SVR. These models were combined with an elastic net to create an ensemble model. The preprocessing methodology included the treatment for missing data and Z-score normalization. Caetano et al.^(14)^ used a data-driven method to predict the hospital LOS for a dataset of 26,431 admissions. The best model was random forest (RF), and the worst models were ordinary least square (OLS) and decision tree (DT). The methodology considered a preprocessing strategy, including k-nearest neighborhood (k-NN) imputation to deal with missing values and Z-score normalization to put the numeric values on the same scale. Moreover, a log transformation was applied to the covariate “previous LOS” and the outcome variable “LOS.”

The most common performance metric used to compare prediction models is the RMSE, followed by the MAE and the coefficient of determination (R^2^). From table 1, we can note that the studies with the best performance were those of Caetano et al.,^(14)^ Muhlestein et al.,^(13)^ and Li et al.^(12)^ The achieved results may be explained by the development of a structured data-driven methodology. These studies were included in the preprocessing step, the treatment for missing data, the log (or Box-Cox) transformation for ICU LOS, and Z-score normalization. Moreover, their methodology included splitting the dataset into training and testing cohorts and using a cross-validation step to analyze the model overfitting. Li et al.^(12)^ also explored new features and analyzed the collinearity between existing features.

Regarding the type of models tested in each study, we can separate them into statistical and data-driven models, as presented in table 2. We note that SVR, a state-of-art data-driven model, overcame the other models in two studies. Other models that presented good results were GBT, RF, GLM, LMM, and LASSO. Therefore, we suggest that future studies consider the following steps to achieve a reasonable prediction for ICU LOS: data extraction and feature engineering; treatment of missing data and outliers; data splitting into training and testing; data preprocessing, including collinearity analysis, feature selection, transformations to resolve skewness and normalization; cross-validation to analyze overfitting; and training, testing and comparing different types of models, including data-driven models. Moreover, future studies should report their results in terms of prediction error (RMSE and MAE), which can help researchers to make conclusions about the best models and make novel recommendations.

**Table 2:** Models included in each prediction study

Author	Statistical models										Data-driven models							
	APACHE model	OLS	GLM	LMM	EGLM	SN	FMM	TE	CPH	LASSO	ANN	k-NN	RF	SVR	RVR	DT	GBT	NB
Moran et al.^(4)^		x	x	x	x	x	x	x										
Verburg et al.^(10)^	x	x	x						x									
Houthooft et al.^(11)^											x	x	x	x	x	x		
Li et al.^(12)^										x								
Muhlestein et al.^(13)^		x									x	x	x	x		x	x	x
Caetano et al.^(14)^		x									x		x	x		x		

APACHE - Acute Physiology and Chronic Health Evaluation; OLS - ordinary least square; GLM - generalized linear model; LMM - linear mixed model; EGLM - extended generalized linear model; SN - skew-normal/skew-t; FMM - finite mixture model; TE - treatment effect; CPH - cox proportional hazard; LASSO - least absolute shrinkage and selection operator; ANN - artificial neural network; k-NN - k-nearest neighbors; RF - random forest;

Regarding the distribution of ICU LOS, most authors tested the log transformation to reduce the distribution skewness. The truncation of ICU LOS data is a common measure to avoid extreme values. Therefore, truncation at high percentiles (95% or 99%) is an alternative to identify outliers. However, truncation can be unfair because there may be substantial differences in the truncated values, and the largest improvements in efficiency may be achieved in patients with the longest ICU LOS. Therefore, we recommend being careful when comparing models using truncated data with models using original data.

Clearly, no single model should be used in all situations. The best result will depend on each dataset, and the models should be trained specifically for each case. Moreover, it is crucial to extract the relevant covariates from the ICU database. Studies have demonstrated that there is a nonlinear relation between ICU LOS and patient severity. In other words, more severe patients tend to have a longer LOS. However, the sickest patients are also those at higher risk of death, which may decrease the expected ICU LOS. Therefore, it is important to include features related to patient severity. Peres et al.^(15)^ suggested a list of risk factors for ICU LOS that should be included in prediction models (e.g., comorbidities, invasive interventions, laboratory markers, and main reasons for ICU admission). The data-driven models will be able to understand this nonlinear relationship if relevant features are included in the analysis. Including irrelevant variables can increase the dimensionality of the problem, which may disturb the model results. On the other hand, excluding relevant features in advance may generate suboptimal results. Therefore, the extraction of variables from the dataset should be done with caution.

Our study has some limitations. First, the work of Houthooft et al.^(11)^ and Li et al.^(12)^ analyzed restricted ICU populations. Houthooft et al.^(11)^ examined a cohort including only medical patients, while Li et al.^(12)^ investigated a single ICU cohort. Second, we included two articles that focused their analysis on hospital LOS prediction instead of ICU LOS. The distribution of hospital LOS may be similar to that of ICU LOS; however, some assumptions may be different from each other. Third, one article^(11)^ made the ICU LOS prediction on day 5 instead of at admission. The distribution of ICU LOS after day 5 was not the same compared to the original LOS, which may affect the comparison analysis.

## CONCLUSION

Although predicting intensive care unit length of stay can be valuable for several stakeholders (e.g., clinicians, patients, families, and administrators), currently, most published models present limitations on individual LOS or overall intensive care unit performance evaluation. Future studies to derive and validate intensive care unit length of stay models should include in their tests data-driven models, especially those developed for large datasets. In addition, these models should be displayed in near real-time and in user-friendly platforms to allow information use at the point of care that can positively impact clinical outcomes.
